# Aberrant Protein Turn-Over Associated With Myofibrillar Disorganization in FHL1 Knockout Mice

**DOI:** 10.3389/fgene.2018.00273

**Published:** 2018-07-23

**Authors:** Jingjing Ding, Yan Fei Cong, Bo Liu, Jianing Miao, Lili Wang

**Affiliations:** Medical Research Center of Shengjing Hospital, China Medical University, Shenyang, China

**Keywords:** FHL1, protein turn-over, autophagy, myopathy, myofibrillar disorganization

## Abstract

Mutations in the FHL1 gene, and FHL1 protein deletion, are associated with rare hereditary myopathies and cardiomyopathies. FHL1-null mice develop age-dependent myopathy and increased autophagic activity. However, the molecular pathway involved in contractile function and increased autophagic activity in the FHL1-null mouse has not yet been fully elucidated. In this study, FHL1 protein was knocked out in mice using Transcription Activator-like Effector Nucleases (TALENs) and the IRS1-FOXO1/mTOR signaling pathway was investigated in skeletal muscles and heart. TALEN constructs caused targeted mutations in 30% of newborn mice; these mutations caused a deletion of 1–13 base pairs which blocked synthesis of the full-length FHL1 protein. Furthermore, 2.5-month old FHL1-null male mice were not prone to global muscular fatigue when compared with WT littermates, but histological analysis and ultrastructural analysis by transmission electron microscopy confirmed the presence of myofibrillar disorganization and the accumulation of autophagosome or autolysosome-like structures in FHL1-null mice. Moreover, autophagy and mitophagy were both activated in FHL1 KO mice and the degradation of autophagic lysosomes was impeded. Enhanced autophagic activity in FHL1 KO mice was induced by FOXO1 up-regulation and protein synthesis was increased via mTOR. The cytoskeletal proteins, MYBPC2 and LDB3, were involved in the formation of pathological changes in FHL1 KO mice. Markers of early differentiation (MEF2C and MYOD1) and terminal differentiation (total MYH) were both up-regulated in tibialis anterior (TA) muscles in FHL1 KO mice. The number of type I and type II fibers increased in FHL1-null TA muscles, but the number of type| | b, and type | | d fibers were both reduced in FHL1-null TA muscles. The results obtained from the heart were consistent with those from the skeletal muscle and indicated autophagic activation by FOXO1 and an increase in protein synthesis via mTOR also occurred in the heart tissue of FHL1 knockout mice. In conclusion, aberrant protein turn-over associated with myofibrillar disorganization in FHL1 knockout mice. the up-regulation of FOXO1 was associated with enhanced autophagic activity and pathological changes in the muscle fibers of FHL1 KO mice. These results indicated that autophagy activated by FOXO1 is a promising therapeutic target for hereditary myopathies and cardiomyopathies induced by FHL1.

## Introduction

The *Fhl1* gene is located on chromosome Xq36, and encodes FHL1A (also known as Slim1, KyoT1 or transcript variant 3 in the mouse), FHL1B (Slimmer, KyoT3 or transcript variant 1), and FHL1C (KyoT2 or transcript variant 4). The FHL1A isoform can also be transcribed from an alternative upstream start codon, producing a transcript with 48 additional nucleotides or 16 extra amino acids at the N terminus of the protein (transcript variant 2 in the mouse) (Chan et al., 1998; Lee et al., 1998). FHL1 is a multifunctional protein and northern blot analysis has confirmed the high expression of FHL1 in skeletal muscle and heart. Consequently, an increasing number of studies have focused on the function of FHL1 in skeletal muscle or heart development. Thus far, a number of FHL1 mutations have been identified, resulting in at least six different X-linked myopathies or cardiomyopathies (Wilhelmsen et al., 1996; Quinzii et al., 2008; Schessl et al., 2008, 2009; Windpassinger et al., 2008; Schoser et al., 2009). Loss of the FHL1 protein can cause muscle hypertrophy, reduced subcutaneous fat, rigid spine, short stature and further extends the phenotype of FHL1-related myopathies (Willis et al., 2016). In order to evaluate the effects of FHL1 loss-of-function on skeletal muscle development and homeostasis, an FHL1-null mouse model was used and observed reduced survival rates in FHL1-null-mice, associated with age-dependent impairment of muscle contractile function and a significantly lower capacity for exercise. Furthermore FHL1-null mice developed age-dependent myopathy and increased autophagic activity (Domenighetti et al., 2014). However, the molecular pathways involved in contractile function and increased autophagic activity in the FHL1-null mouse has not yet been fully elucidated.

The size of skeletal myofibers and contractile function are influenced by the turnover of skeletal muscle proteins; the myofiber phenotype has also been associated with the contractile function of muscle (Schiaffino et al., 2013). Studies have shown that the IGF 1–Akt signal pathway regulates protein synthesis via mTOR, and protein degradation occurs via transcription factors of the FoxO family (Matsuzaki et al., 2005; Tzivion et al., 2011; O’Neill et al., 2016; Le Plénier et al., 2017). The FoxO1 upstream signals including IGFs, insulin, and IRS regulate the transcriptional activity of FoxO1 by phosphorylating FoxO1 in a PI3K-Akt-dependent manner. Phosphorylated FoxO1 is excluded from the nucleus and thus loses its capacity to bind to target regulatory elements (Matsuzaki et al., 2005; Tzivion et al., 2011; O’Neill et al., 2016; Le Thanh et al., 2017). In muscle, FoxO1 has been implicated in the control of both proteasomal and autophagy-lysosomal degradation pathways (Milan et al., 2015). Class I HDACs are key regulators of FoxO and the mechanisms underlying muscle-atrophy during both nutrient deprivation and skeletal muscle disuse (Beharry et al., 2014). Furthermore, FoxO1 inhibited the gene expression of slow fibers (MyHC I) by down-regulating MEF2C expression, thus increasing the transcriptional activation of slow fiber genes (Xu et al., 2017). Therefore, FoxO1 plays important roles in muscle protein degradation pathways and myofiber-type determination in normal muscle development. However, the function of FoxO1 in the FHL1-null mouse have not yet been fully elucidated. Therefore, in the present study, the *Fhl1* gene was knocked out using Transcription Activator-like Effector Nucleases (TALENs), and the function of IRS1-FoxO1 and mTOR in muscle protein turn-over and differentiation was investigated in *Fhl1* gene knockout mice.

## Materials and Methods

### Ethics Statement

All animals in this study were from the Animal Center of Shengjing Hospital at China Medical University. All studies were performed in accordance with the protocol approved by the Institutional Animal Care and Use Committee of the China Medical University (NO.2013PS06K). All surgery was performed under anesthesia, and all efforts were made to minimize suffering.

### Generation of FHL1-Null Mice

C57B6 mice were kept in an animal room under SPF conditions (temperature: 24 ± 1°C; humidity: 55–60%; photoperiod: 12 h light/12 h dark) with free access to food and water. We used Transcription Activator-like Effector Nucleases (TALENs) to knockout the *Fhl1* gene. Mice *Fhl1* genomic DNA sequences were collected from the GenBank Database^1^. TALEN plasmids were constructed by the Golden Gate method and confirmed by DNA sequencing analysis. *Fhl1*-TALEN mRNAs were prepared using the mMESSAGE mMACHINE T7 Ultra kit (Ambion) according to the manufacturer’s instructions and diluted to a working concentration of 50 ng/μL in injection buffer (10 mM Tris, 0.1 mM EDTA, pH 7.4). C57BL/6 (B6) mouse strains were used as embryo donors and foster mothers, respectively. Female B6 mice (7–8 weeks old) were super-ovulated by intraperitoneal injections of 5 IU of pregnant mare serum gonadotropin (PMSG; Sigma–Aldrich) and 5 IU of human chorionic gonadotropin (hCG; Sigma–Aldrich) at 48-h intervals. The super-ovulated female mice were then mated with B6 stud males, and fertilized embryos were collected from oviducts. Fifty ng of TALEN mRNAs was then injected into the cytoplasm of fertilized eggs with well-recognized pronuclei in M2 medium (Sigma–Aldrich) using a piezo-driven micromanipulator (Prime Tech). After incubation at 37°C for 24 h, two-cell embryos were selected and transferred into the oviducts of pseudopregnant foster mothers to obtain live pups. Genomic DNA was extracted from the F0 generation the tail, and a 1,350 bp PCR product was obtained using the PCR sense primer: 5′-acctgccctgcccaatagtcttt-3′ and the antisense primer 5′-agcccaagcctgaaatccaaaag-3′. PCR products from the F0 generation mouse tails were purified, cloned, and sequenced to screen the positive founder mice with a FHL1 protein frame shift. The positive F0 generation mice were then crossed with C57BL/6J mice.

### Genotyping and Genomic DNA Sequencing of Offspring Mice With a 13 bp Deletion

Genomic DNA was isolated from the tail tips of offspring mice with a 13 bp deletion, using the DNeasy Blood and tissue Kit (QIAGEN, Mannheim, Germany), following the manufacturer’s instructions. Primers for mice genotyping were designed around the deleted base pairs and primer sequences were as follows: P1: 5′-ATGAATCTCCCAGAATCCC-3′; P2: 5′-GAATACACCTGAAGCCCAC-3′ (wild type product length = 584 bp; *Fhl1* KO product length = 571 bp). Amplification was accomplished with Premix Taq (Takara RR902Q) and PCR amplifications were performed on an Bio-Rad PCR machine under specified conditions: 94°C for 3 min, and 31 cycles each of 94°C for 30 s, 54°C for 30 s, 72°C for 60 s, and 72°C for 10 min. PCR products were separated and visualized on 2% agarose gels. PCR products amplified from each genomic DNA were sequenced and then the genotype of the individual was confirmed.

### Mouse Exercise Protocol

Five pairs of 2.5-month-old FHL1-null and WT male littermates were subjected to a single round of a treadmill running protocol. Mice were placed in their respective lanes and allowed to adjust to their surroundings for 5 min. During the adjustment period, the treadmill was set to 5 m/min. Every 15 min, the speed of the treadmill was ramped up in 3 m/min steps from the initial 5 m/min. The treadmill incline was slowly adjusted to 12°from the initial 0° and every 15 min, the treadmill incline rose by 3°. Mice were allowed to run until they were exhausted, defined as the inability to continue running despite 20 s of continuous mild electrical stimulation. The time and distance run was recorded for each mouse.

### Hematoxylin and Eosin Staining of Skeletal and Heart Muscle

Five pairs of 2.5-month-old FHL1-null and WT male littermates were used in this part of the study. Oxidative and mixed muscles (gastrocnemius, Gas), and glycolytic muscles (tibialis anterior, TA; and triceps, Tri) and heart were dissected and then fixed in 4% paraformaldehyde, embedded with paraffin, and cut into 5 mm thick cross-sections. The remaining muscles were quickly frozen in liquid nitrogen for the subsequent detection of protein and RNA expressions.

### Transmission Electron Microscopy

Five pairs of 2.5-month-old FHL1-null and WT male littermates were used in this part of the study. Detailed steps were accomplished according to previous study (Domenighetti et al., 2014). At least 50 electron micrographs per genotype were examined in each muscle at different magnifications using a transmission electron microscope (Hitachi 7100).

### Measurement of Fiber-Type

Transitions in myofiber phenotype in TA of FHL1-null mice were examined by real-time quantitative RT-PCR. Primers for specific MHC used in the PCR were shown in **Table 1**. Three pairs of 2.5-month-old FHL1-null and WT male littermates were used in this study. Total RNA was isolated from TA and RNA (1 μg) was reverse-transcribed with oligo(dT) primer and SuperScript II (Takara); 1/5 of the complementary DNA (cDNA) mixture served as a template for subsequent PCR. Real-time PCR was performed on a Roche 480 PCR system with SYBR Green (Takara). The housekeeping gene, GAPDH was used as an endogenous control and the relative levels of gene expression for each sample were calculated using the 2^-ΔΔct^ method.

**Table 1:** Primers for specific MyHC using in Q-PCR.

Gene	Sequences	Length
Gapdh	ATGTTTGTGATGGGTGTGAA	122 bp
	ATGCCAAAGTTGTCATGGAT	
MyHC I	AGTCCCAGGTCAACAAGCTG	144 bp
	TTCCACCTAAAGGGCTGTTG	
MyHC IIa	AGTCCCAGGTCAACAAGCTG	129 bp
	GCATGACCAAAGGTTTCACA	
MyHC IIb	TGCCAAGTCCATCCCGAAGT	234 bp
	GGTCACCCGCATCAACCAGC	
MyHC IIX	AGTCCCAGGTCAACAAGCTG	112 bp
	CACATTTTGCTCATCTCTTTGG	

### Western Blotting

Total proteins, or cytoplasmic and nuclear proteins, were extracted from frozen muscles using Minute^TM^ total protein extraction kit (SD-001; Invent Biotech, INC) and Minute^TM^ cytoplasmic and nuclear extraction kit (SC-003; Invent Biotech, INC) containing Roche Complete Protease Inhibitor Cocktail and 1 mM PMSF. Protein concentrations were determined using the BCA method (Thermo Fisher). Protein extracts were subjected to SDS-PAGE electrophoresis and visualized with SuperSignal West Pico Chemiluminescent substrate (Pierce). Primary antibodies used in immunoblots were as follows and against: GAPDH (KangChen Bio-tech: KC-5G4-1:10000), FHL1 (Sigma: WH0002273M1-1:2000), FoxO1 (Millipore: 05-1075-1:500), phosphorylated FoxO1 (Santa: sc-16561-R-1:50), acetylated FoxO1 (Santa: sc-49437-1:50), LC3B (CST: #2775-1:1000), BNIP3 (Abcam: AB10433-1:1000), Beclin1 (Abcam: AB10433-1:500), P62 (Sigma–Aldrich: p0067-1:500), IRS1 (CST: #3194-1:1000), mTORC (CST: #2983-1:500), P70-S6 K (CST: #2708-1:1000), phosphorylated P70-S6K (CST: #9234-1:1000), MYOD1 (CST: #13812-1:500), MEF2C (CST: #5030-1:500]), MYH (Santa: sc-12117-1:100), LDB3 (Thermo Fisher: PA5-30434-1:1000]), and MYBPC2 (Abnova: PAB19214-1:2000).

### Statistical Analyses

All graphs represent the mean ± standard error of the mean (SEM). A two-tailed two sample Student’s *t*-test of unequal variance was performed using GraphPad software to compare the means of the wildtypes and FHL1 KO groups.

## Results

### Generation of Homozygous *Fhl1* Knockout Mice With TALENs

**FIGURE 1:**
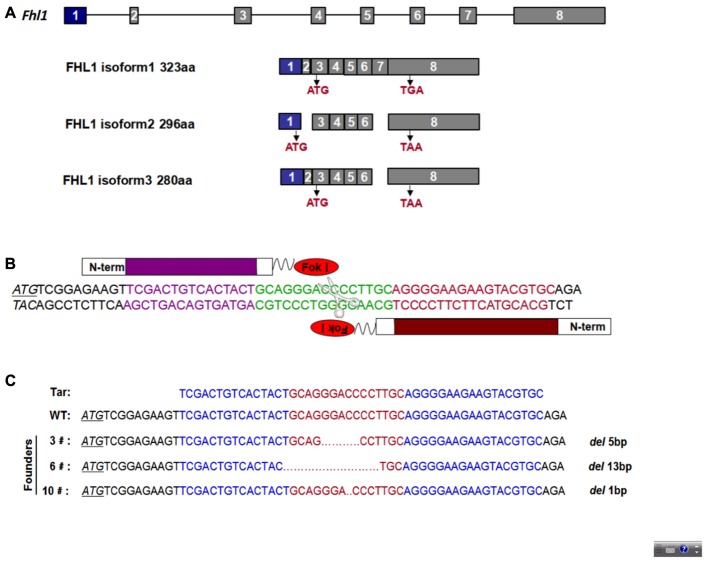
TALEN-mediated *Fhl1* mutant mice. **(A)** Structural representation of the *Fhl1* gene showing different transcripts and homologous protein isoforms. **(B)** DNA-binding sequences (in red or purple) and the spacer region for Fhl1-TALEN. **(C)** DNA sequences of the *Fhl1* locus from live F0 mice.

To design an appropriate TALEN targeting site, a sequence was targeted at the ATG code within the third exon of *Fhl1* (Gene ID: 14199). *Fhl1*-TALEN-L1 was designed against the sequence 5′-TCGACTGTCACTACT-3′ for the sense strand, and *Fhl1*-TALEN-R1 was designed against the sequence 5′-GCACGTACTTCTTCCCCT-3′ for the antisense strand. The targeted sequences were separated by a spacer region of 15 bp (GCAGGGACCCCTTGC; **Figures 1A,B**). All 140 fertilized eggs continued to survive after being individually injected with 50 ng/μL of *Fhl1*-TALEN mRNA. Two-cell embryos were transferred into the oviducts of 7 pseudopregnant foster mothers and two pseudopregnant foster mothers (28.57%) became pregnant. Three founders were found in the 10 newborns (30%; see **Table 2**). Founders were genotyped by genomic DNA sequencing and the three founder genotypes were shown in **Figure 1C**.

**Table 2:** TALEN-mediated *Fhl1* gene targeting in C57BL/6J mice.

Dose (ng/μl)	Injected zygotes	Survived zygotes	2-cell embryos	Transferred embryos	Recipients	Pregnant recipients	Newborns	Founders
50	140	140 (100%)	140	140	7	2 (28.57%)	10 (7.14%)	3 (30.00%)

### Genotyping and Genomic DNA Sequencing of Offspring Mice With a 13 bp Deletion

**FIGURE 2:**
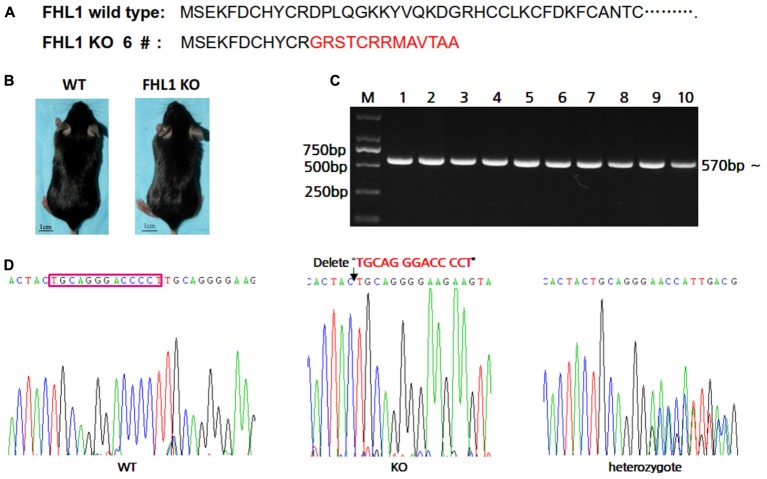
Generation of TALEN-mediated *Fhl1* mutant mice. **(A)** FHL1 protein in a wild type mouse and #6 founder mouse. Only 24 amino acids were produced and degraded quickly in the FHL1 knockout (KO) mouse. **(B)** The general appearance of wild type and FHL1 KO mice. No overt developmental anomaly was found in the FHL1 KO mice. **(C)** Electrophoresis results of expected PCR products from genomic DNA. **(D)** The results of a 13 nucleotide deletion from genomic DNA sequencing.

In order to easily design primers for PCR genotyping 6#F0 founder with a 13 bp deletion were chosen for further study. The effects of deletion on *Fhl1* genomic DNA sequences and FHL1 protein are shown in **Figure 2A**; only 24 amino acids were produced and degraded quickly in the FHL1 knockout mice. The general appearance of FHL1 KO mice were shown in **Figure 2B**. Primers for genotyping were designed and PCR products were amplified from mice with different genotypes (**Figure 2C**). Mouse genome DNA sequencing results are shown in **Figure 2D**.

### Enhanced Autophagic Activity in FHL1-Null Muscles

**FIGURE 3:**
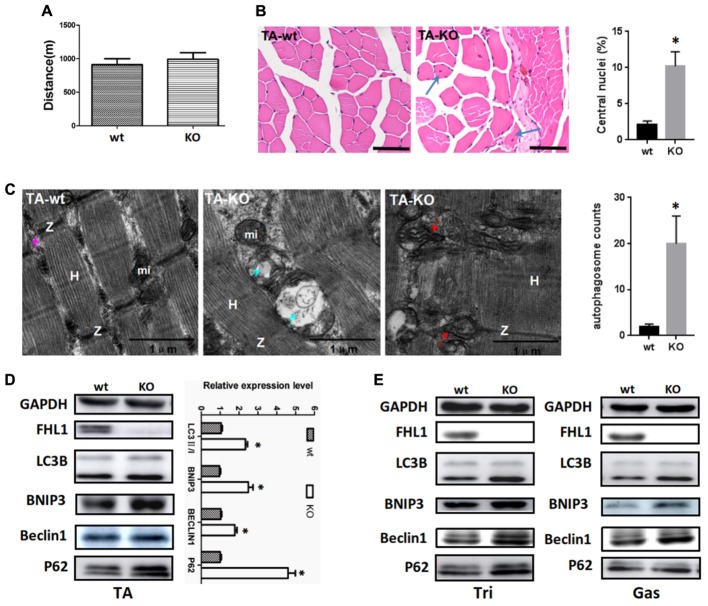
Enhanced autophagic activity in FHL1-null muscles. **(A)** The result of treadmill tests on 2.5-month-old male mice. A fatigue exercise protocol performed on treadmills showed that FHL1-null mice were not prone to global muscular fatigue when compared with wild type (WT) littermates. **(B)** Histological analysis of the tibialis anterior (TA) muscles of WT and FHL1-null male mice and the presence of atrophic and hypertrophic fibers and an increased percentage of fibers with centralized nuclei in FHL1-null male mice; the arrow points to fibers with centralized nuclei. Quantitation of the number of muscle fibers with central nuclei in TA was represented as % of the total number of muscle fibers analyzed. Data were shown as the mean ± SEM. *^∗^P* < 0.05. Scale bar: 50 μm. **(C)** Transmission electron microscopy of TA muscles; the blue arrow points to autophagosome or autolysosome-like structures while the red arrow points to extensive sarcomere (S) and sarcoplasmic reticulum (SR) disarray. The numbers of autophagosome were counted in WT and FHL1-null TA. Six electron micrographs per genotype were examined and data were shown as the mean ± SEM. *^∗^P* < 0.05. **(D)** Western blot analysis of markers for autophagy and mitophagy in 2.5-month-old WT and FHL1-null tibialis anterior (TA). Statistical analysis indicated that the LC3-II/LC3-I ratio, BINP3 (mitophagy marker), and BECLIN1 (autophagy marker) were all significantly increased in FHL1-null muscle. However, there was also evidence of p62 accumulation, which indicated autophagy and mitophagy were both activated and that the degradation of autophagic lysosomes was impeded in FHL1 KO muscles. Data are shown as the mean ± SEM. ^∗^*P* < 0.05. **(E)** Western blot analysis of markers for autophagy and mitophagy in 2.5-month-old WT and FHL1-null triceps muscle (Tri) and gastrocnemius (Gas) muscles.

A fatigue exercise protocol performed on treadmills showed that FHL1-null male mice were not prone to global muscular fatigue when compared with WT littermates (**Figure 3A**). Histological analysis of the tibialis anterior muscles (TA) of WT and FHL1-null male mice confirmed marked variability in myofiber size, the presence of atrophic and hypertrophic fibers and an increased percentage of fibers with centralized nuclei in FHL1-null male mice (**Figure 3B**). Ultrastructural analyses by transmission electron microscopy of the tibialis anterior muscles showed the accumulation of autophagosome or autolysosome-like structures in FHL1-null mice. Furthermore, FHL1-null mice also showed disarranged sarcomeric Z-lines and enlargement and branching of the sarcoplasmic reticulum (**Figure 3C**). Gene expression associated with autophagy (Beclin-1), mitophagy (Bnip3), autophagic flux (increased LC3-II/LC3-I ratios and a lack of p62 accumulation) were also investigated; gene expression data showed that Beclin-1 Bnip3, LC3-II/LC3-I ratios were all increased in oxidative and mixed muscles (gastrocnemius, Gas), and glycolytic muscles (tibialis anterior, TA; and triceps, Tri) in FHL1 KO mice. At the same time, p62 accumulation was clearly evident (**Figures 3D,E**). These results indicated that compared with FHL1-WT mice, autophagy and mitophagy were both activated in the FHL1 KO mice receiving normal food and drink, and the degradation of autophagic lysosomes was impeded.

### The Up-Regulation of FOXO1 Was Associated With Enhanced Autophagic Activity in the Muscles of FHL1-Null Mice

**FIGURE 4:**
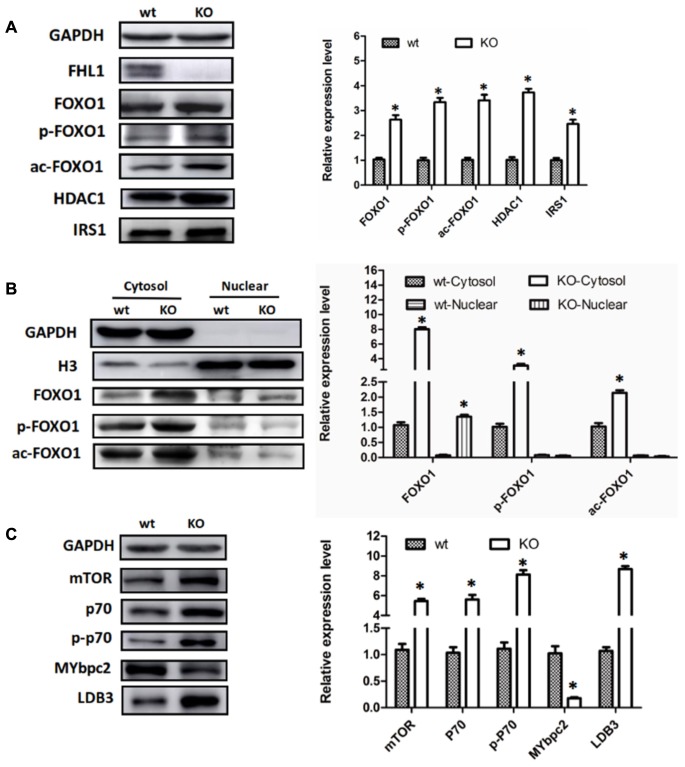
Aberrant protein turn-over in FHL1-null mice. **(A)** Western blot analysis of FOXO1 expression in TA muscles, along with its upstream molecules, HDAC1 and IRS in 2.5-month-old WT and FHL1-null TA muscles. **(B)** Subcellular localizations of total FOXO1, along with acetylated and phosphorylated FOXO1. **(C)** Protein synthesis via mTOR was increased in FHL1-null TA muscles. Data are shown as the mean ± SEM. ^∗^*P* < 0.05.

Because FOXO1 plays important roles in the pathways involved in muscle autophagy-lysosomal degradation pathways, we decided to investigate FOXO1 expression in TA muscles, along with its upstream molecules, HDAC1 and IRS. The results showed that FOXO1, HDAC1, and IRS1 were all up-regulated in the TA of FHL1 KO mice (**Figure 4A**). Moreover, post-translational modifications (acetylation and phosphorylation) of FOXO1 were also activated in the TA of FHL1 KO mice. Acetylated and phosphorylated FOXO1 would have been excluded from the nucleus and thus would lose its capacity to bind to its target regulatory elements. Because of this, we decided to investigate the subcellular localization pattern of total FOXO1, along with acetylated and phosphorylated FoxO1. Data showed that although FOXO1 was excluded from the nucleus via acetylation and phosphorylation, the FOXO1 existing in the nucleus was still up-regulated in FHL1 KO mice (**Figure 4B**). This indicated that FOXO1 up-regulation was associated with enhanced autophagic activity in the TA of FHL1 KO mice. In addition, upstream of FOXO1, HDAC1 plays an important role in muscle atrophy and its up-regulation in the TA of FHL1 KO mice further confirmed that HDAC1-FOXO1 participated in pathological changes in FHL1 KO mice.

### Protein Synthesis via mTOR Was Increased in the Muscles of FHL1-Null Mice

In the TA of FHL1 KO mice, mTORC1 was up-regulated and its effectors, p70S6 kinase 1 (p70S6K1) was activated. The phosphorylation of p70S6K1 was also up-regulated compared with the TA of FHL1 WT mice (**Figure 4C**). These results suggested that protein synthesis was increased in FHL1-null muscles via the mTOR pathway. Moreover, the abnormal expression of cytoskeletal proteins, MYBPC2 and LDB3 (**Figure 4C**), were detected in the TA, which indicated that cytoskeletal proteins participated in the formation of pathological changes in FHL1 KO mice.

### Skeletal Muscle Differentiation Was Regulated by MEF2C in the TA Muscle of FHL1-Null Mice

**FIGURE 5:**
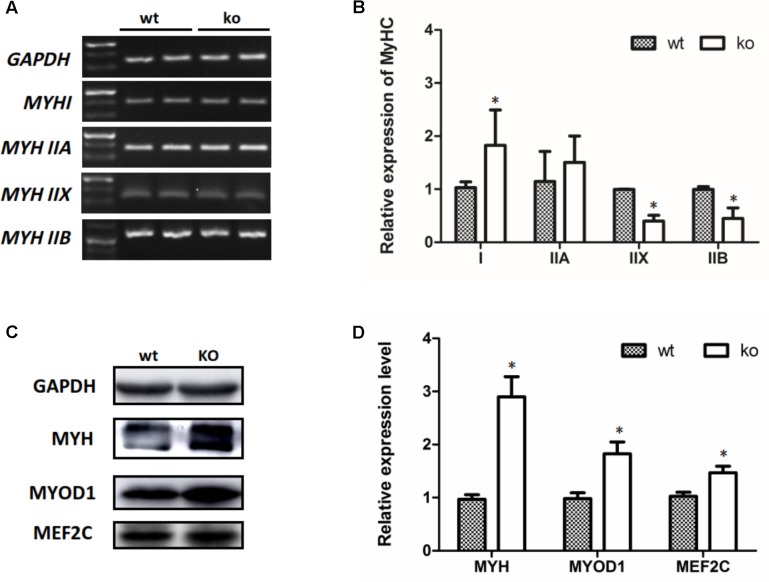
The myofiber phenotype transitions in FHL1-null mice. **(A)** The electrophoresis result of PCR products of specific MHC. **(B)** The relative levels of specific MHC. MYH| and MYH| | a expression was increased in FHL1-null TA muscles, while MYH| | b and MYH| | d both showed decreased expression in FHL1-null TA muscles. **(C)** Western blot analysis of markers of skeletal muscle differentiation. **(D)** Relative expression levels of markers of skeletal muscle differentiation. Markers of early differentiation (MEF2C and MYOD1) and terminal differentiation (total MYH) were both upregulated in FHL1-null TA muscles. Data are shown as the mean ± SEM. ^∗^*P* < 0.05.

Transitions in the myofiber phenotype in the TA of FHL1-null mice were investigated by real-time quantitative RT-PCR. PCR products of specific MHC were separated by 2% agarose gels (**Figure 5A**). The relative levels of specific MHC are shown in **Figure 5B**. These results indicated that MYH I and MYH IIa expression were increased in FHL1-null TA muscles while that of MYH|| b and MYH|| d were both decreased (**Figure 5B**). Markers of early differentiation (MEF2C, MYOD1) and terminal differentiation (total MYH) were both shown to be up-regulated (**Figures 5C,D**). MEF2C can increase the transcriptional activation of slow fiber (MYH |) genes. Consequently, its up-regulation resulted in myofiber phenotype transitions in the TA muscles of FHL1-null mice.

### Autophagic Activation by FOXO1 and Protein Synthesis Was Increased in the Heart Tissue of FHL1-Null Mice via the mTOR Pathway

**FIGURE 6:**
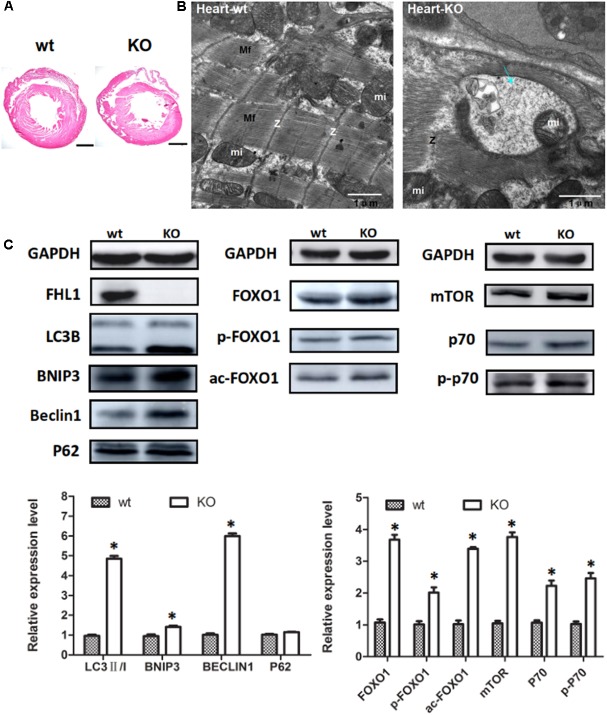
Enhanced autophagic activity and aberrant protein turn-over in the heart of FHL1-null mice. **(A)** Hematoxylin and eosin staining of the heart of wild type and FHL1 KO mice. Scale bar: 1000 μm. **(B)** Transmission electron microscopy of the heart; the blue arrow points to autophagosome or autolysosome-like structures. **(C)** Western blot analysis of markers for autophagy/mitophagy, total FOXO1, along with acetylated and phosphorylated FOXO1 and mTOR signaling pathway in heart in 2.5-month-old WT and FHL1-null mice. Data are shown as the mean ± SEM. ^∗^*P* < 0.05.

Because the FHL1 protein is highly expressed in the heart, we decided to investigate the heart phenotype in FHL1-knockout mice. Data suggested that the FHL1-null heart developed in a similar manner to WT mice (**Figure 6A**). However, TEM confirmed that the heart of FHL1-knockout mice contained autophagosome or autolysosome-like structures and mis-arranged sarcomeric Z-lines (**Figure 6B**). Beclin-1, Bnip3, and LC3-II/LC3-I ratios were all increased in the hearts of FHL1 KO mice. Furthermore, there was clear evidence of p62 accumulation (**Figure 6C**). Moreover, FOXO1 and HDAC1 were both upregulated in the heart of FHL1 KO mice (**Figure 6C**). The mTORC1 was upregulated, and its effector, p70S6 kinase 1 (p70S6K1), and the phosphorylation of p70S6K1, were upregulated in the heart of FHL1 KO mice compared with FHL1 WT mice (**Figure 6C**). These results indicated that autophagic activation by FOXO1 and protein synthesis increased via the mTOR pathway in the heart tissue of FHL1 KO mice.

## Discussion

FHL1 localizes to the sarcomere and the sarcolemma and is believed to participate in muscle growth and differentiation, as well as in sarcomere assembly. Recent studies have indicated that mutations in four and-a-half LIM domain protein 1 (FHL1) are associated with rare hereditary myopathies, including reducing body myopathy (RBM), X-Linked myopathy with postural muscle atrophy (XMPMA), Emery-Dreifuss muscular dystrophy (EDMD), and cardiomyopathies (Kley et al., 2016; Xue et al., 2016; Ehsan et al., 2017; Pillar et al., 2017; Le Thanh et al., 2017). All these hereditary myopathies exhibit clinical features of muscular dysfunction including progressive muscular weakness, postural muscle atrophy and muscle wasting and weakness (Schessl et al., 2011). In 2016, a study reported a male with a deletion of the entire FHL1 gene who was 15 years of age and presented with muscle hypertrophy, reduced subcutaneous fat, a rigid spine, and a short stature. This extended the phenotype of FHL1-related myopathies and should prompt future testing in undiagnosed patients who present with unexplained muscle hypertrophy, contractures and a rigid spine, particularly if the patient is male (Willis et al., 2016). Collectively, these findings suggested that mutations in the *Fhl1* gene (mutational FHL1 protein), or deletion of the entire *Fhl1* gene (complete FHL1 protein deletion) could both result in a muscle phenotype, but the molecular mechanism involved in muscle phenogenesis has not yet been fully elucidated. In the mouse model, inactivation of the FHL1 has no baseline phenotype; however, mice lacking FHL1 lack a response to pressure overload in the heart (Sheikh et al., 2008). Furthermore, FHL1-null mice develop age-dependent myopathy and increased autophagic activity (Domenighetti et al., 2014). Decreased survival rates in FHL1-null mice are associated with an age-dependent impairment of muscle contractile function (Domenighetti et al., 2014). However, the molecular pathway involved in contractile function and increased autophagic activity in the FHL1-null mouse has not been elucidated.

Skeletal muscles make up at least 40% of the body mass and the cytosol of muscle cells is filled by contractile proteins which determine the cellular size and morphology. Therefore, protein turnover can significantly affect muscle mass and performance (Gottlieb and Carreira, 2010; Rüegg and Glass, 2011; Lyon et al., 2013; Anthony, 2016). Over the last few years, increased autophagy-lysosome activity has been found to be associated with muscle atrophy, which suggested that regulating the level of autophagy may represent a good therapeutic target for many myopathies and cardiomyopathies (Bargiela et al., 2015; Dowling et al., 2015; Li et al., 2015; Tham et al., 2015; Kang and Ji, 2016). In this study, the *Fhl1* gene was knocked out using TALENs in mice and an overt pathological phenotype was not observed in 2.5 month-old FHL1 KO mice. Although no overt pathological abnormalities were seen in paraffin sections, the abnormalities may have been missed because of the lack of histological studies in frozen muscle tissue. However, TEM and western blotting confirmed that autophagosome or autolysosome-like structures and an autophagic activation was present in the TA of FHL1 KO mice. These results were consistent with a previous study (Domenighetti et al., 2014). Moreover, a marker of mitophagy (Bnip3) was also found to be increased in TA, Tri, Gas muscles, which suggested that mitophagy might be responsible for autophagic activation in FHL1 KO mice, at least in part.

Insulin has also been shown to control the synthesis and degradation of muscle proteins, and after stimulation with their respective ligand, IR (the insulin receptor) activated common downstream molecular pathways, including the IRS/PI3K/Akt pathway (Bonaldo and Sandri, 2013; O’Neill et al., 2016). Akt controls protein synthesis via mTOR and protein degradation via transcription factors of the FoxO family. In muscle, FoxO1 has been implicated in the control of both proteasomal and autophagy-lysosomal degradation pathways (Milan et al., 2015). FoxO1 transgenic mice show markedly reduced muscle mass and fiber atrophy, further supporting the notion that FoxO proteins are sufficient to promote muscle loss (Kamei et al., 2004). In the present study, IRS1 (insulin receptor substrate 1) and Foxo1 were both increased in the TA of FHL1 KO mice. FoxO activity is regulated by several different posttranslational modifications, including phosphorylation and acetylation (Wimmer et al., 2015; Li et al., 2016). We therefore decided to study the subcellular localization patterns of total FoxO1, and acetylated and phosphorylated FoxO1. Although increased levels of phosphorylated and acetylated FoxO1 were detected in the cytoplasm of FHL1 KO mice, FoxO1 in the nucleus was upregulated. This indicated that FoxO1 up-regulation was associated with enhanced autophagic activity in the TA of FHL1 KO mice. A recent study revealed a key role for histone deacetylases (HDACs) in the control of skeletal muscle homeostasis and autophagic flux (Moresi et al., 2012). HDAC1 activates FoxO and is both sufficient and required for skeletal muscle atrophy (Beharry et al., 2014). In addition, our results indicated that HDAC1 up-regulation also played an important role in muscle atrophy in the TA of FHL1 KO mice.

The mTOR is a crucial kinase located downstream of insulin and nutrient-sensitive pathways required for cell growth. The mTORC1 promotes protein synthesis predominantly through the phosphorylation of two key effectors, p70S6 kinase 1 (p70S6K1) and eIF4E binding protein (4EBP) (Demontis and Perrimon, 2010; Fry et al., 2011; Tang et al., 2014; Saxton and Sabatini, 2017). In the TA of FHL1 KO mice, mTORC1 was up-regulated and its effectors, p70S6K1 and the phosphorylation of p70S6K1, were up-regulated compared with the TA of WT mice. These results suggested that protein synthesis via mTOR was increased in FHL1-null muscles. Moreover, abnormal expression of the cytoskeletal proteins, MYBPC2 and LDB3 were detected in the TA of FHL1 KO mice. Mutations and abnormal expression of MYBPC2 or LDB3 have been associated with myopathies and cardiopathies (Li et al., 2010; Meurs and Kuan, 2011), which indicates that cytoskeletal proteins participate in the formation of pathological changes in FHL1 KO mice.

The myofiber phenotype transitions in FHL1-null mice were examined and the results indicated that the numbers of type I and type IIa fibers were increased in FHL1-null TA muscles, while numbers of type Ib and type IId fibers were both decreased. Markers of early differentiation (MEF2C and MYOD1) and terminal differentiation (total MYH) were both upregulated. MEF2C increased the transcriptional activation of slow fiber (MYH I) genes. Thus, its up-regulation resulted in myofiber phenotypic transitions in FHL1-null mice TA muscles. Histological analyses showed that the over-expression of FoxO1 in skeletal muscle reduced the size and number of type I and type II fibers (Kamei et al., 2004). Xu et al. (2017) reported that FoxO1 inhibited slow fiber gene expression (MyHC | ) by down-regulating MEF2C expression (Xu et al., 2017). In this study, FoxO1 and MEF2C were both activated in the TA of FHL1-null mice, which indicated that other molecules are involved in the regulation of MEF2C expression and might play a vital role in the TA of FHL1-null mice.

Because FHL1 protein is expressed at high levels in the heart, we decided to investigate the heart phenotype in FHL-1 knockout mice. The primary results obtained from heart tissue were consistent with those of skeletal muscle and indicated autophagic activation by FoxO1 and an increase in protein synthesis via the mTOR pathway in the heart tissue of *Fhl1* knockout mice. Dysregulation in the activity of FoxO1 has been implicated in the pathophysiology of diabetic cardiomyopathy (DCM) (Battiprolu et al., 2012; Kandula et al., 2016), along with ischemic and cardiac hypertrophy (Ni et al., 2006), which suggests that FoxO1 is a therapeutic target for these myocardial diseases.

## Conclusion

Mutations in the *FHL1* gene, along with deletion of the FHL1 protein, are associated with rare hereditary myopathies and cardiomyopathies. In this study, FHL1 protein was knocked out using TALENs in mice. FoxO1 up-regulation was associated with enhanced autophagic activity and pathological changes of muscle fibers in FHL1 KO mice, which indicated the activation of autophagy by FoxO1, and therefore represents a promising therapeutic target for hereditary myopathies and cardiomyopathies induced by FHL1.

## Author Contributions

JD and LW: experimental design. JD, YC, BL, and JM: experiments operation. JD and LW: data curation. LW: funding acquisition. JD and LW: writing original draft.

## Conflict of Interest Statement

The authors declare that the research was conducted in the absence of any commercial or financial relationships that could be construed as a potential conflict of interest.

## Footnotes

1 https://www.ncbi.nlm.nih.gov/gene/14199
